# Effect of Oral and Topical Sodium Bicarbonate on Functional Recovery and Soccer-Specific Performance After Exercise-Induced Muscle Damage: A Randomized, Double-Blind, Placebo-Controlled Study

**DOI:** 10.3390/nu17213383

**Published:** 2025-10-28

**Authors:** William H. Gurton, Lewis A. Gough, Anthony Lynn, Mayur K. Ranchordas

**Affiliations:** 1 Sport and Physical Activity Research Centre, Health Research Institute, Sheffield Hallam University, Sheffield S10 2BP, UK; m.ranchordas@shu.ac.uk; 2 Research for Human Performance and Health Lab, College of Life Sciences, Birmingham City University, Birmingham B42 2LR, UK; lewis.gough@bcu.ac.uk; 3 Food and Nutrition Subject Group, Sheffield Business School, Sheffield Hallam University, Sheffield S1 1WB, UK; t.lynn@shu.ac.uk; 4 Advanced Wellbeing Research Centre, Sheffield Hallam University, Sheffield S9 3TU, UK

**Keywords:** ergogenic aids, vertical jump, agility, repeated sprints, muscle soreness, extracellular buffering

## Abstract

**Objectives**: This study assessed the influence of oral and topical sodium bicarbonate (SB) on recovery and soccer-specific performance after exercise-induced muscle damage (EIMD). **Methods**: In a randomized, double-blind, placebo-controlled, parallel group design, 24 soccer players were allocated to oral SB, topical SB (PR Lotion, Momentous), or placebo (PLA) (*n* = 8 per condition) and attended six laboratory sessions: (i) familiarization, (ii) baseline measures, and (iii) four experimental trials on consecutive days. Muscle damage was induced on day 1 using 40 × 15 m sprints, after which either 0.3 g·kg^−1^ body mass (BM) SB (SB-ORAL), 0.9036 g·kg^−1^ BM PR Lotion (SB-LOTION), or an equivalent PLA was given. Recovery outcomes were measured post-EIMD, 1 d, 2 d, and 3 d post (day 1–4). Soccer-specific performance was repeated 3 d post, with supplements administered again 2 h pre-exercise. Recovery measures included muscle soreness, vertical jump height, and maximal voluntary isometric contraction. Illinois agility test (IAT), 8 × 25 m repeated sprints, and Yo-Yo Intermittent Recovery Test Level 2 (Yo-Yo IR2) were assessed as soccer-specific performance. **Results**: Neither SB-ORAL nor SB-LOTION accelerated indices of recovery but decline in soccer-specific performance from baseline to 3 d post was attenuated for SB-ORAL, with significant effects for IAT (*p* = 0.032, *g* = 1.69) and Yo-Yo IR2 (*p* = 0.026, *g* = 1.61) compared with PLA. **Conclusions**: SB did not accelerate recovery following EIMD but prescribing oral SB before subsequent exercise might rescue key performance indicators. These findings offer implications for practitioners working with soccer players during periods where full recovery is not achieved.

## 1. Introduction

Sustained bouts of strenuous exercise, such as repeated sprint efforts, that are a common requirement during soccer matches may cause muscle damage and inflammation [1,2,3]. Exercise-induced muscle damage (EIMD) is greatest when physical tasks involve repetitive eccentric muscle contractions where muscles lengthen under tension [4]. Soccer players repeatedly perform intense eccentric movement tasks (e.g., sprints, deceleration, changes in direction) throughout the course of a match, leading to intracellular muscle damage that subsequently impairs muscle function and causes delayed onset muscle soreness [1,2,3]. Factors like declining intramuscular pH and disturbances to ionic balance that contribute towards skeletal muscle fatigue also negatively influence performance in latter stages of matches [5,6]. Impairments in physical performance and EIMD symptoms persist up to 5 d after strenuous exercise [7,8]; however, competitive soccer matches can be separated by as little as 3 d (i.e., periods of fixture congestion), meaning insufficient time exists for recovery. As a consequence, players begin to perform worse in each match, whilst the cumulative effect of inadequate recovery from one week to another leads to fatigue accumulation and a greater predisposed risk of injury [9].

Research has explored whether nutritional supplements accelerate recovery after EIMD [1,2,3,10], but one that has received less focus is sodium bicarbonate (SB) [11]. SB is an extracellular buffer that elevates blood bicarbonate (HCO_3_^−^) and protects against intramuscular acidosis during exercise by increasing efflux of hydrogen ions (H^+^) from muscles [12]. Evidence suggests SB improves soccer-specific performance like repeated sprint times and Yo-Yo Intermittent Recovery Test Level 2 (Yo-Yo IR2) distance [13,14,15] and accelerates short-term recovery [16,17], although benefits across longer time periods are unclear. Scientific rationale for why SB might improve recovery following EIMD could be underpinned by elevated acid-base balance mitigating initial metabolic deficiencies (e.g., declining muscle pH) that increase the susceptibility of muscle fibres to the mechanical stresses of muscle damaging exercise [18]. SB has also reduced cellular stress response (e.g., heat shock protein-72 (HSP72)) and oxidative stress up to 24 h post-exercise [19,20,21,22] and augmented the expression of peroxisome proliferator-activated receptor-γ coactivator 1-alpha (PGC-1α) during recovery from exercise [23]. Collectively, this offers mechanistical support for why SB could attenuate cellular damage and inflammation post-EIMD [7,8] and promote muscle regeneration needed to preserve functional capacity throughout recovery and soccer-specific performance 3 d later [24]. To date, only Battazza et al. [25] have examined the effect of SB on recovery after EIMD, reporting no benefits 1 d post; however, the authors failed to explore whether benefits existed up to 3 d later—considered the key timeframe for optimizing performance during competitive soccer. This prompts the need for further work examining the application of SB to accelerate recovery.

A body of scientific work has examined the effectiveness of analgesic muscle lotions (e.g., Biofreeze, ibuprofen gels) for attenuating muscle soreness and accelerating functional recovery (i.e., countermovement jump (CMJ), maximal voluntary isometric contraction (MVIC)) after EIMD [26,27,28]. These either act by delivering potent non-steroidal anti-inflammatory drugs to a localized site through the skin [29], or by using menthol—a cyclic terpene alcohol produced from mint oils—to create cooling sensations via activation of the transient receptor potential melastatin-eight (TRPM8) family of ion channels that lessens perception of pain [26]. Recently, a topical SB muscle cream (PR Lotion) that is used in a similar manner to analgesic muscle gels has been investigated [13,14,30]. This was developed to deliver SB into systemic circulation through the transdermal route [31] and contains small amounts of menthol. Equivocal findings exist for increases in blood buffering capacity and performance after PR Lotion [13,30,32], but Gibson et al. [32] showed positive changes in serum pH (+0.04 au). There is a strong rationale for why PR Lotion could be a better option than oral SB for accelerating recovery. Topical SB eliminates concerns about gastrointestinal (GI) distress that limits application of the supplement [33], and is more practically feasible than ingesting large number of capsules (~25–30 for 80 kg athletes) needed to achieve ergogenic doses for oral SB. Moreover, PR Lotion may induce strong localized cooling sensations [14] that reduce an individuals’ perception of muscle soreness compared to their actual degree of muscle damage [26,28].

It is not yet known whether oral or topical SB improve functional recovery and subsequent performance after EIMD in soccer players. During periods of fixture congestion in soccer, practitioners seek interventions that improve recovery and offset decline in performance before a second match [1,34]. Anecdotal evidence from soccer teams suggests some practitioners use PR Lotion as a recovery aid, but scientific support is lacking. Rigorous placebo-controlled studies are needed to ensure evidence-based practices are being followed. Therefore, the aim of this study was to examine the effect of oral and topical SB supplementation on functional recovery and soccer-specific performance after EIMD.

## 2. Materials and Methods

### 2.1. Participants

Thirty soccer players (21 males, 9 females) were allocated to oral SB (SB-ORAL), topical SB (SB-LOTION), or placebo (PLA). Sample size was based on a priori power calculation using a mixed-methods analysis of variance (ANOVA) approach. Based on effect size (partial eta squared, *η_p_*^2^ = 0.175) for treatment differences in CMJ height post-EIMD from a similar study [35], expected correlation between repeated measures (*r* = 0.75), and target power of 0.80 with two tailed α level set at 0.05, it was estimated that 9 participants per condition were needed to observe significant effects. CMJ was used as it is a sensitive test for detecting reductions in neuromuscular function after EIMD [36]. To account for dropouts, 10 participants were allocated to each condition, with sex initially balanced (i.e., 7 men, 3 women). Six men dropped out (*n* = 4, time commitments; *n* = 2, lost to follow up), and one woman withdrew due to injury, with a male participant recruited as a replacement (*n* = 24 completed the study; Table 1). Our cohort of soccer players were classified as Tier 2 graded athletes [37], who played soccer weekly recreationally or in competitive leagues. Participants were excluded if they (i) used supplements (e.g., SB, polyphenols) or commercial analgesic muscle lotions for recovery, (ii) had any intolerances to corn flour or PR Lotion, and/or (iii) were diagnosed with medical or musculoskeletal conditions that impacted their ability to perform exercise. The study was performed in accordance with the revised Declaration of Helsinki and approved by the Institutional Ethics Committee (ER66612740). All participants provided written informed consent before enrolment.

### 2.2. Study Design

**Figure 1:**
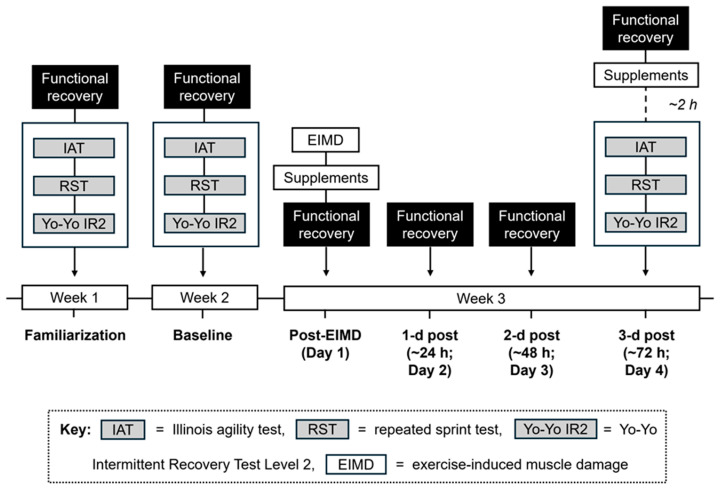
Experimental schematic of testing timeline/protocol. Participants randomized to oral SB, topical SB, or placebo. Functional recovery included soreness, general wellbeing, CMJ, DJ, and MVIC. Supplements given post-EIMD (day 1) and 3 d post (day 4; ~2 h pre-performance tests).

A randomized, double-blind, placebo-controlled, parallel-group study design was used to investigate the effect of oral and topical SB on indices of recovery and soccer-specific performance after EIMD. Participants were randomly assigned to one of three conditions in blocks of three (#1, #2, #3; then #4, #5, #6, etc.) by a laboratory technician not involved with data collection. The number of females in each condition was initially matched to account for sex-specific differences in anaerobic performance after SB supplementation [38], but one dropped out of PLA. This randomized allocation approach ensured that the principal investigator remained blinded to the condition each participant received and helped minimize variability in primary outcomes (e.g., CMJ height, repeated sprint times) across conditions. Testing was conducted across a 3 week period (Figure 1). Muscle damage was induced on day 1 of the experimental test week using a repeated sprint running task (40 × 15 m sprints, with 5 m deceleration). Functional recovery measures were taken over four consecutive days (post-EIMD, 1 d, 2 d, 3 d post). Supplements were given after the muscle damaging exercise and again 3 d post (2 h before participants repeated soccer-specific performance outcomes). This protocol was based off practices from fixture congestion in professional soccer where recovery interventions are prescribed after a match—before being repeated daily or once 3 d post—in an attempt to restore performance prior to the second match (~72 h later) [1,34].

Dependent variables evaluated as part of the functional recovery protocol were muscle soreness, general wellbeing score, vertical jump height (CMJ and drop jump (DJ)), and knee extensor MVIC. These represent attributes that players seek to quickly restore after competition [26]. Illinois agility test (IAT), 8 × 25 m repeated sprints, and Yo-Yo IR2 were assessed as soccer-specific performance outcomes in line with previous research [13,14,15].

### 2.3. Procedures, Familiarization and Baseline Measures

Participants attended 6 separate laboratory test sessions for familiarization, baseline, and experimental measures. Testing took place at a similar time of day (~2 h) to minimize cofounding effects of diurnal variation [39]. Participants were instructed to avoid vigorous exercise, alcohol, caffeine, and foods with a high potential renal acid load 24 h before testing [40]. They were also asked to wear the same clothing and footwear for all testing sessions. Compliance to pre-experimental controls was checked by the investigator.

During familiarization, physical characteristics (stature, body mass) were recorded using an ultrasonic measuring station (SECA, Hamburg, Germany). Thereafter, participants were familiarized to functional recovery and performance tests. Two vertical jump techniques (CMJ, DJ) were carried out using an optoelectrical system (Optojump, Microgate, Italy) to measure lower-body power. During CMJ efforts, participants stood with feet shoulder-width apart, hands on hips, flexed their knees and hips into a partial squat, and, without stopping, exploded upwards with maximal force. For DJ efforts, participants stepped off a 30 cm high box, landed on both feet, and jumped vertically as high as possible whilst minimizing their ground contact time. Three maximal efforts were completed for each (30 s between efforts), with average height recorded to the nearest 0.1 cm. Lower-body muscle strength was measured via unilateral knee extension MVIC using an isokinetic dynamometer (Cybex NORM^®^, Humac, PR, USA). Seat position was adjusted for each individual to align the lateral epicondyle of their non-dominant knee with the dynamometer axis of rotation. First, participants completed a warm-up protocol consisting of progressive efforts (50%, 75%, 90% maximum). Next, they performed three MVIC attempts, each lasting 3 s and being separated by 60 s. Peak and average torque were recorded to the nearest newton-metre (nm). Participants moved to the indoor sports hall and underwent a soccer-specific warm up comprising light jogging, 20 m shuttles, vertical jumps, IAT circuits, 10 m sprints, and self-selected stretches [13,14]. Upon completion, participants carried out three IAT circuits and the 8 × 25 m repeated sprint test. These were interspersed by 3 min, with repeated effects separated by 30 s. Light gates (Brower Timing Systems, Draper, UT, USA) were placed at the start/end. Average times were calculated to the nearest 0.01 s. Lastly, participants performed a Yo-Yo IR2, requiring them to repeatedly carry out 2 × 20 m shuttle runs at increasing speeds dictated by audio signals [41]. Each stage was separated by 10 s recovery where participants jogged around a cone 5 m behind the start line. Test termination was classified as consecutive failures to reach the finish before the signal, at which point distance (last completed stage) was recorded.

Participants returned to the laboratory/indoor sports hall for baseline measures of all dependent variables. On arrival, general wellbeing and muscle soreness questionnaires were completed. To assess general wellbeing—a popular recovery measure in soccer—validated psychological questionnaires based off recommendations from Hooper & Mackinnon [42] were completed. This provided an aggregate score for subjective feelings of fatigue, sleep quality, general muscle soreness, stress levels, and mood using five-point scales (scores of 1 to 5; least, to most, positive) [43]. Visual analogue scales anchored from “0 mm” (no soreness) to “200 mm” (unbearable pain) were used to measure muscle soreness of the quadriceps and gastrocnemius [44]. First, participants squatted to 90° knee flexion, held the position for 3 s, and rated the degree of soreness in their quadriceps. Next, they raised their heels as much as possible, balanced on their toes for 3 s, and rated the soreness in their gastrocnemius. Aggregate muscle soreness was calculated from the sum of these scores. Participants then carried out the vertical jump and MVIC tests before undertaking the soccer-specific warm-up and performance tests.

### 2.4. Exercise-Induced Muscle Damage Task

A running-based task known to induce muscle damage [26,45] was used as it is more ecologically valid in soccer players than other methods (e.g., isokinetic dynamometry) [10]. The protocol comprised an initial warm-up adapted from previous research [26]. This started with 5 laps around the perimeter of the indoor sports hall where participants performed soccer-specific movement patterns (jogging, high knees, cariocas, side steps). Participants completed four 20 m sprints progressing from ~50% to ~70%, ~90%, and ~100%. They also engaged in self-selected stretches for 2 min. Next, forty 15 m maximal sprints comprising a 5 m deceleration zone were performed (taking ~3 s to complete). Every sprint was separated by 30 s. Combined duration of warm-up and the protocol was ~30 min. Given the exploratory nature of this study, an EIMD task was chosen to replicate intense eccentric movement patterns of soccer that induce muscle damage [1,2,3], thus allowing us to assess the scope for future work exploring more ecologically valid scenarios.

### 2.5. Nutritional Supplements

Depending on randomization, participants received either (i) 0.3 g·kg^−1^ body mass (BM) SB in capsules and 0.9036 g·kg^−1^ BM placebo lotion (SB-ORAL), (ii) placebo capsules and 0.9036 g·kg^−1^ BM PR Lotion (SB-LOTION), or (iii) placebo capsules and 0.9036 g·kg^−1^ BM placebo lotion (PLA). Supplements were prepared by a laboratory technician. Size 0 vegetarian capsules (Your Supplements, Stockport, UK) were filled with ~0.8 g SB (Health Leads Ltd., Llandysul, UK) or ∼0.4 g corn flour (Sainsbury’s, London, UK). Corn flour was used as the oral placebo, as it is an inert substance that blinds SB [46]. PR Lotion was provided from Momentous (Park City, UT, USA). The placebo-matched lotion had SB removed but still contained 0.5% menthol as per commercial PR Lotion. A 0.9036 g·kg^−1^ BM PR Lotion dosage accounts for the proportion of SB (33.2%) per gram of PR Lotion to match a 0.3 g·kg^−1^ BM oral SB dose [13,14,30].

### 2.6. Experimental Testing Week (Day 1–4)

Participants attended 4 sessions across consecutive days. Upon arriving at the laboratory on day 1, general wellbeing questionnaires were completed before 95 µL capillary blood samples were collected into blood gas tubes (Vitrex Medical, Herlev, Denmark) and analyzed for acid–base balance (pH, HCO_3_^−^) using an i-STAT Alinity (Abbott, Maidenhead, UK). Participants then performed the repeated sprint running muscle damage task. Completion time of each 15 m sprint was recorded to the nearest 0.01 s using light gates. Acid–base balance was measured immediately post-EIMD. Thereafter, participants received the first dose of their allocated supplement. These were administered ~10 min after the EIMD protocol. Capsules were consumed alongside carbohydrate food (1.5 g·kg^−1^ BM; cornflakes with milk, energy gels, cereal bars) and 7 mL·kg^−1^ BM water as three equal doses at 15 min intervals across 30 min. Lotion was applied to participants’ entire legs and lower back as three 0.3012 g·kg^−1^ BM doses at the same time-points. Participants performed the post-EIMD functional recovery protocol concomitantly such that the second/third doses of capsules and lotion were given upon completion of the vertical jump and MVIC tests. Questionnaires for blinding efficacy (as per previous research [46]) and cooling sensation were completed post-supplementation (~40 min post-EIMD). Aggregate scores for cooling sensation of the quadriceps and calves were evaluated using 7-point Likert scales [14].

Functional recovery outcomes and acid–base balance were measured on day 2 (1 d post; ~24 h), day 3 (2 d post; ~48 h), and day 4 (3 d post; ~72 h). After measures had been collected on day 4, participants repeated the cooling sensation questionnaire. Visual analogue scales (“0” mm to “100” mm; no symptom to severest) were filled out to quantify aggregate GI distress from eight side-effects (nausea, flatulence, abdominal discomfort, gut fullness, bowel urgency rating, diarrhea, vomiting, belching) [17]. Next, blood lactate was analyzed using a Biosen C-Line (EKF Diagnostics, Cardiff, UK), and the same supplements from day 1 were given over 30 min. Participants rested for a further 85 min before carrying out the warm-up and performance testing battery (TB). Timeframes between supplementation and tests were IAT (+120 min), 8 × 25 m repeated sprints (+130 min), and Yo-Yo IR2 (+140 min). These were based on evidence that peak changes in blood pH and HCO_3_^−^ occur ~120–150 min after SB ingestion [32,47]. Cooling sensation, blinding efficacy, and GI questionnaires were repeated post-supplementation, pre- and post-TB. Acid–base balance was measured pre- and post-TB, with lactate measured after the warm-up and each of the soccer-specific tests (IAT, 8 × 25 m repeated sprints, Yo-Yo IR2).

### 2.7. Statistical Analysis

All data analyses were conducted using SPSS (version 26.0). Shapiro–Wilk tests were utilized for assessing whether standardized residual data were normally distributed. Mauchly’s tests of sphericity were used to check homogeneity of variance for all ANOVA analyses, and where necessary, violations of the assumption were corrected using the Greenhouse–Geisser adjustment. To determine test–retest reproducibility, typical error of measurement (TEM) and intraclass-correlation coefficients (ICC) were calculated between familiarization and baseline data. Two-way mixed-model, absolute agreement ICCs were interpreted as poor (*r* < 0.50), moderate (*r* = 0.50–0.75), good (*r* = 0.75–0.90), or excellent (*r* > 0.90) [48]. One-way analysis of variances (ANOVA) were used to explore significant differences between conditions at baseline and change in soccer-specific performance. Significant condition effects were examined using Tukey HSD tests. Two-way mixed ANOVA (between-subject factor: condition) were performed to assess time course changes in dependent variable. The number of time levels (within-subject factor) were as follows: indices of recovery (five: baseline, post-EIMD, 1 d, 2 d, and 3 d post), general wellbeing (six; extra measurement point pre-EIMD), acid-base balance (seven: pre-EIMD, post-EIMD, 1 d, 2 d, and 3 d post, pre-TB, post-TB), aggregate cooling sensations (six: post-EIMD, post-supplement day 1 (post-supp-1), pre-supplement day 4 (pre-supp-4), post-supplement day 4 (post-supp-4), pre-TB, post-TB), and aggregate GI distress (four: pre-supp-4, post-supp-4, pre-exercise, post-exercise). *η_p_*^2^ was reported as the effect size for significant ANOVA main effects and interactions. These were interpreted using the classifications of 0.01, 0.06, and 0.14 as small, medium, and large, respectively [49]. Effect sizes for changes in soccer-specific performance were presented as Hedges’ g (g) [50] and interpreted as trivial (<0.20), small (0.20–0.49), moderate (0.50–0.79), or large (≥0.80) [49]. Data are presented as mean ± SD, with 95% *CI* reported for significant differences in performance. Statistical significance was set at *p* < 0.05.

## 3. Results

### 3.1. Baseline Measures and Test–Retest Reproducibility

Data for baseline dependent variables are shown in Table 2. No significant differences were observed between conditions (*p* > 0.05).

Excellent reproducibility was shown for CMJ height (TEM = 5.4%, ICC = 0.94), DJ height (TEM = 6.8%, ICC = 0.92), and MVIC torque (TEM = 10%, ICC = 0.96). Soccer-specific performance was also reproducible, with moderate-to-good reproducibility for average IAT (TEM = 3.57%, ICC = 0.69) and 8 × 25 m repeated sprint (TEM = 2.51%, ICC = 0.89) times, and excellent reproducibility for Yo-Yo IR2 distance (TEM = 12%, ICC = 0.98).

### 3.2. Repeated Sprint Muscle Damage Protocol

Sprint performance data from the muscle damage protocol are presented in Table 3. There were no significant differences between conditions for average, fastest, or total sprint time, and a similar decrement score was reported (*p* > 0.05). This confirmed that participants exerted themselves to a similar degree, and, therefore, the extent of muscle damage could be expected to be similar between conditions.

### 3.3. Vertical Jump Height

**Figure 2:**
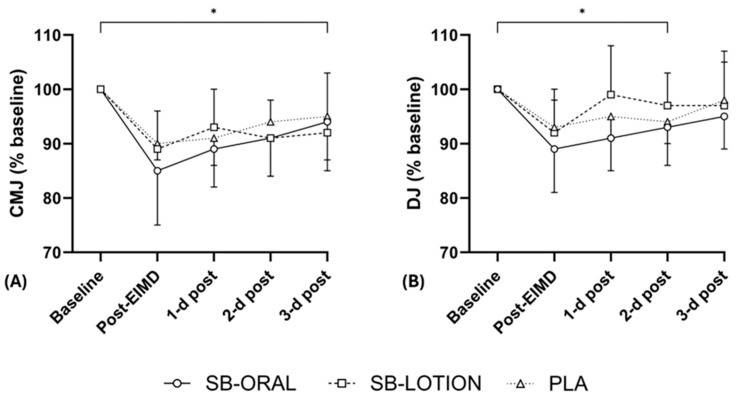
Mean ± SD countermovement jump (CMJ; (**A**)) and drop jump (DJ; (**B**)) height throughout testing expressed as a % of baseline values. * depicts significant time effects (*p* < 0.05) vs. baseline irrespective of condition.

Condition × time interactions for CMJ were not statistically significant for maximum (*F*(8, 84) = 0.609, *p* = 0.768) or average (*F*(8, 84) = 0.976, *p* = 0.460) height. Main effects of time existed for maximum (*F*(4, 84) = 19.573, *p* < 0.001, *η_p_*^2^ = 0.482) and average (*F*(4, 84) = 21.359, *p* < 0.001, *η_p_*^2^ = 0.504) height. Condition*time interactions for DJ were not statistically significant for maximum (*F*(8, 84) = 0.892, *p* = 0.527) or average (*F*(8, 84) = 0.650, *p* = 0.733) height. Main effects of time existed for maximum (*F*(4, 84) = 5.982, *p* < 0.001, *η_p_*^2^ = 0.222) and average (*F*(4, 84) = 8.758, *p* < 0.001, *η_p_*^2^ = 0.294) height. Independent of condition, average CMJ and DJ height remained less than baseline by 3 d post. Jump height expressed as a % of baseline values is presented in Figure 2.

### 3.4. Maximal Voluntary Isometric Contraction

Condition × time interactions were not statistically significant for peak MVIC (*F*(5.729, 60.159) = 0.393, *p* = 0.874) or average MVIC (*F*(5.424, 56.957) = 0.462, *p* = 0.817) torque. Main effects of time existed for peak (*F*(2.865, 60.159) = 8.805, *p* < 0.001, *η_p_*^2^ = 0.295) and average (*F*(2.712, 56.957) = 8.923, *p* < 0.001, *η_p_*^2^ = 0.298) torque.

### 3.5. Muscle Soreness and Wellbeing Score

**Figure 3:**
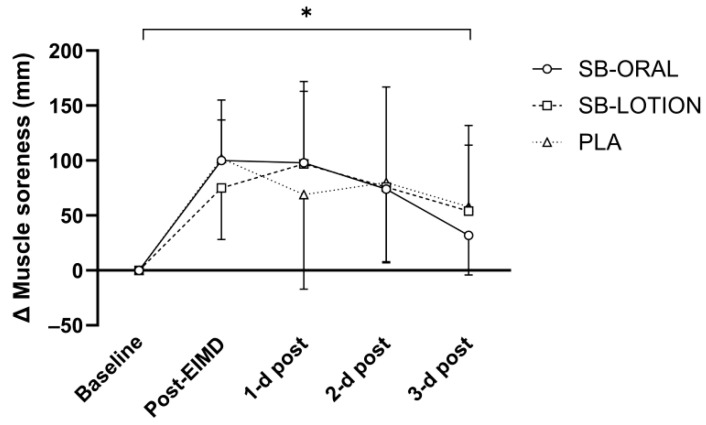
Mean ± SD change (Δ) in aggregate muscle soreness (quadriceps and calves). * depicts significant time effects (*p* < 0.05) vs. baseline irrespective of condition.

Condition × time interactions were not statistically significant for aggregate muscle soreness (*F*(8, 84) = 0.319, *p* = 0.957) or wellbeing score (*F*(7.050. 74.023) = 0.607, *p* = 0.750). Main effects of time existed for aggregate muscle soreness (*F*(4, 8) = 16.185, *p* < 0.001, *η_p_*^2^ = 0.435) and wellbeing score (*F*(3.525, 74.023) = 7.502, *p* < 0.001, *η_p_*^2^ = 0.263). Independent of condition, aggregate muscle soreness had not returned to baseline by the end of recovery. Aggregate muscle soreness expressed as change (Δ) from baseline is shown in Figure 3.

### 3.6. Soccer-Specific Performance

**Figure 4:**
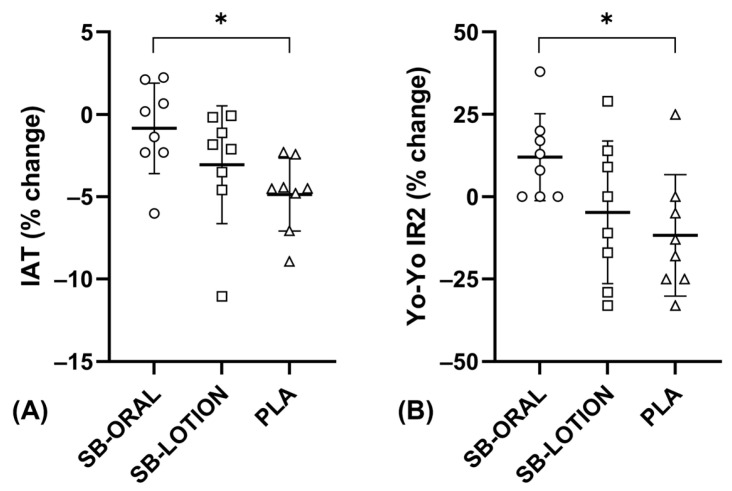
Individual changes in Illinois agility test (IAT; (**A**)) and Yo-Yo Intermittent Recovery Test Level 2 (Yo-Yo IR2; (**B**)) performance between conditions from baseline to 3 d post. Mean change (%) displayed as a bold horizontal line. * *p* < 0.05 for SB-ORAL vs. PLA.

Baseline performance data are shown in Table 2. Agility time 3 d post was similar to baseline for SB-ORAL (17.19 ± 0.81 s) but slower for SB-LOTION (17.73 ± 1.60 s) and PLA (17.95 ± 0.95 s). Changes in agility times from baseline were statistically different between conditions (*F*(2, 21) = 3.766, *p* = 0.040, *η_p_*^2^ = 0.26). The drop-off in agility time was attenuated for SB-ORAL (−0.14 ± 0.46 s) vs. PLA (−0.83 ± 0.35 s). Hedges g revealed a large effect of SB-ORAL (−0.69 s; 95% *CI*: −1.33, −0.05; *p* = 0.032; *g* = 1.69). Repeated sprint time 3 d post was similar to baseline for SB-ORAL (4.01 ± 0.21 s) but slower for SB-LOTION (4.18 ± 0.36 s) and PLA (4.20 ± 0.28 s). Changes in sprint times from baseline were not statistically different between conditions (*F*(2, 21) = 2.100, *p* = 0.147). Yo-Yo IR2 distance 3 d post was higher than baseline for SB-ORAL (470 ± 269 m), similar for SB-LOTION (430 ± 275 m), and lower for PLA (375 ± 171 m). Changes in Yo-Yo IR2 distance from baseline were statistically different between conditions (*F*(2, 21) = 4.002, *p* = 0.034, *η_p_*^2^ = 0.28). Distance covered during Yo-Yo IR2 improved for SB-ORAL (+45 ± 45 m) and decreased for PLA (−50 ± 70 m). Hedges g revealed a large effect of SB-ORAL (95 m; 95% *CI*: 10, 180; *p* = 0.026; *g* = 1.61). Individual values for % change from baseline to 3 d post for IAT and Yo-Yo IR2 performance are shown in Figure 4.

### 3.7. Acid-Base Balance and Lactate

**Figure 5:**
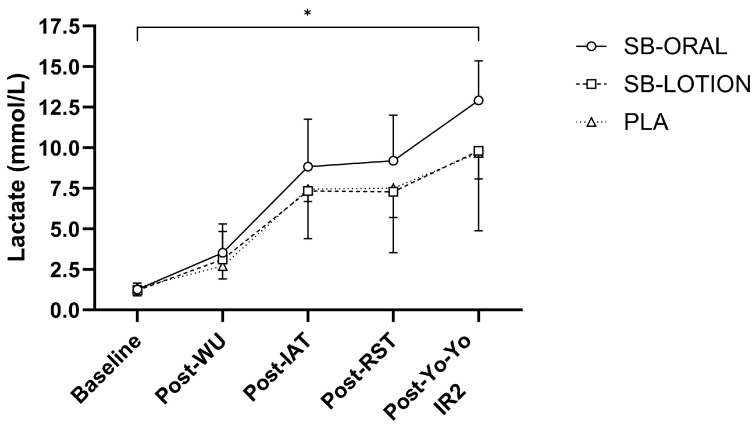
Mean ± SD blood lactate response from baseline to post-exercise on day 4. Abbreviations: WU = warm-up, RST = 8 × 25 m repeated sprint test. * depicts significant time effects (*p* < 0.05) vs. baseline irrespective of condition.

Significant condition × time two-way interactions were shown for blood pH (*F*(5.732, 60.184) = 4.117, *p* = 0.002, *η_p_*^2^ = 0.282) and HCO_3_^−^ (*F*(7.065, 74.188) = 3.953, *p* = 0.001, *η_p_*^2^ = 0.274) but not lactate (*F*(5.334, 56.003) = 1.545, *p* = 0.187). A main effect of time existed for blood lactate (*p* < 0.001, *η_p_*^2^ = 0.868) (Figure 5). Blood pH and HCO_3_^−^ were similar between conditions post-EIMD (*p* > 0.05). At 1 d post, blood pH was higher for SB-ORAL vs. SB-LOTION (+0.02 au; *p* = 0.042) and PLA (+0.02 au; *p* = 0.022), but HCO_3_^−^ did not differ between conditions throughout the recovery period (Table 4; *p* > 0.05). Blood pH and HCO_3_^−^ were elevated pre-soccer TB for SB-ORAL vs. SB-LOTION (+0.06 au, +3.2 mmol·L^−1^; *p* < 0.001, *p* = 0.020) and PLA (+0.05 au, +4.9 mmol·L^−1^; *p* = 0.002, *p* = 0.001). Post-TB, blood pH was elevated for SB-LOTION vs. PLA (+0.06 au; *p* = 0.035) and for SB-ORAL vs. PLA (+0.07 au; *p* = 0.017), with HCO_3_^−^ higher for SB-LOTION vs. PLA (+3.3 mmol·L^−1^; *p* = 0.04).

### 3.8. Gastrointestinal Distress, Cooling Sensations, and Blinding

Condition × time interactions were not statistically significant for GI score (*F*(6, 63) = 0.173, *p* = 0.983), and there was no main effect of time (*F*(3, 63) = 1.880, *p* = 0.142). There were no condition × time interactions for cooling sensations (*F*(6.217, 65.278) = 0.231, *p* = 0.968), but a main effect of time (*F*(3.108, 65.278) = 22.977, *p* < 0.001, *η_p_*^2^ = 0.522) existed. Data for aggregate GI distress and cooling sensations are shown in Table 5.

SB-LOTION and PLA conditions remained blinded throughout the study, with the number of correct guesses less than expected by chance (<33%). Blinding efficacy was less effective for SB-ORAL, with three participants (38%) identifying the condition post-supp-1, post-supp-4, and pre-TB, and five (63%) post-TB. Reasons given for being able to identify SB-ORAL included taste/appearance of capsules, GI side-effects, and improved performance.

## 4. Discussion

The aim of this study was to examine the effect of oral and topical SB supplementation on functional recovery and soccer-specific performance after EIMD. Our repeated sprint protocol was effective at causing muscle damage, with elevated muscle soreness and reductions in gross measures reported as indirect markers of EIMD. No improvements in functional recovery were demonstrated for oral or topical SB compared with placebo, indicating that neither alleviate the detrimental effects of EIMD. However, the decline in IAT and Yo-Yo IR2 performance from baseline to 3 d post was attenuated by prescribing a second oral SB dose prior to the subsequent soccer-specific TB. This suggests that oral SB may rescue key performance indicators in soccer players. These changes in soccer-specific performance were likely attributed to elevated extracellular buffering capacity. Our findings provide implications for practitioners by demonstrating that oral SB ingestion prior to a subsequent exercise bout might recover performance when soccer players have not achieved full recovery. Future research should examine the effect of SB throughout ecologically valid competitive soccer scenarios (e.g., between two matches) before evidence-based recommendations can be made about using SB as a recovery aid.

Neither oral nor topical SB accelerated functional or subjective indices of recovery across a 3 d period following a 40 × 15 m repeated sprint running task. Our work reinforces the limited scientific insight by Battazza et al. [25], in which ingestion of 0.3 g·kg^−1^ BM SB failed to accelerate the restoration of peak torque or attenuate muscle soreness 24 h after an isokinetic dynamometry task. The lack of benefits from oral or topical SB cannot be attributed to our EIMD task causing insufficient muscle damage, as it induced a similar degree of muscle soreness and impairments in gross measures of performance throughout recovery to previous studies [26,45]. One reason why neither SB strategies accelerated recovery could relate to supplementation timing. In contrast to work showing attenuated oxidative stress after SB [19,51], SB was administered post-EIMD. Prescribing oral and topical SB beforehand might have better protected against metabolic deficiencies (e.g., declining intramuscular pH, cellular stress, accumulation of free radicals) that underpin impaired recovery. Our design, however, meant participants could not exert themselves harder during the EIMD protocol (i.e., performance was matched) because of the ergogenic effects from SB [13,14]. Increased workload may have increased participants’ susceptibility to mechanical and cellular stressors that contribute towards muscle damage, thus raising the degree of muscle soreness and subsequently delaying recovery [4,18]. Future research should examine the influence of SB on recovery during ecologically valid soccer scenarios (e.g., attenuation of fatigue between two competitive matches) before conclusions can be drawn regarding its efficacy as a recovery-based ergogenic strategy.

Considering the applied nature of our study, it was not possible to explore underlying physiological mechanisms by which SB may exert its effects on recovery after EIMD. Although neither cellular stress or inflammatory responses were measured, it is said that SB lessens HSP72 expression [19,20,22] and augments regulators of mitochondrial biogenesis (i.e., PGC-1α) [23] during/post-exercise. Reduced cellular damage theoretically means that athletes experience less physiological stress, which, combined with greater muscle fibre regeneration via increased PGC-1α expression after SB, could lead to faster recovery or permit a higher level of subsequent exercise performance [11]. Whilst more work needs to be conducted, it has been suggested that dampened HSP72 expression after SB is attributed to tighter regulation of intracellular acid–base balance [20]. Interestingly, blood pH was higher (~0.02 au) 1 d post for SB-ORAL compared with SB-LOTION and PLA. Despite not translating to improvements in functional recovery, it seems that ingestion of SB restored acid–base balance (hypothetically mitigating cellular stress) throughout the early stages of recovery (~24 h). The importance of accelerated acid–base balance recovery after SB for attenuating functional and biochemical indices of EIMD warrants further investigation.

It has previously been hypothesized that benefits reported for PR Lotion could be underpinned by interactions between menthol and SB that form a protective layer across the skin, augmenting menthol-mediated preservations in force production and reductions in perceived muscle soreness [13]. According to the manufacturer, menthol is a secondary ingredient of PR Lotion, but as large absolute doses are prescribed (~75 g for team sport athletes), the amount of menthol (~0.4 g) is similar to topically applied analgesic gels (e.g., 2 mL of ~3.5% menthol Biofreeze gel) that have been shown to alleviate symptoms of EIMD [28]. Accelerated indices of recovery for menthol-based gels relates to activation of TRPM8 family of ion channels inducing strong cooling sensations that override the noxious A-Delta and C pain fibres, in turn lessening an individuals’ perception of pain and allowing greater effort during a gross motor task [26]. In contrast to a previous study [14], there were no differences in cooling sensation for SB-LOTION. Other than discrepancies in the study design (parallel vs. crossover), equivocal findings could be explained by individual differences in temperature perception. Physiological factors such as age, sex, and body composition contribute towards diversity in thermal perception [52]. Whilst somewhat speculative, certain individuals may report more pronounced cooling sensations after PR Lotion because of greater sensitivity to alterations in skin temperature.

This study was the first to investigate whether oral or topical SB could be effective ergogenic strategies for preventing fatigue-related impairments in performance after EIMD. Topical SB did not offset the drop off from baseline, reinforcing that 0.9036 g·kg^−1^ BM PR Lotion does not improve performance [14,30]. In contrast, oral SB improved agility times and Yo-Yo IR2 distance, such that the decline in performance was attenuated compared with PLA. In fact, 3 d post Yo-Yo IR2 distance was 11% greater than baseline for SB-ORAL. Our results complement existing work showing oral SB to induce small performance benefits for soccer players [13,14,15]. Interestingly, however, the drop-off in repeated sprint time was not significantly different between conditions, which contradicts recent evidence [13,14]. One explanation for contrasting results could relate to sex-specific physiological differences that underpin SB [38]. Previous studies recruited male athletes, who exhibit greater benefits for SB than females [38] due to larger type II muscle fibres that rely more heavily on the glycolytic pathway for adenosine triphosphate production [53]. Due to a small sample size of females, it was not possible to confirm whether sex-dependent differences influenced the effect of SB, but researchers exploring SB in females have reported equivocal results [12,47,54]. In light of our findings, oral SB ingestion could be an effective strategy for rescuing key soccer-specific performance indictors following EIMD; however, future work needs to compare the benefits of SB in men and women throughout soccer-specific exercise to ensure evidence-based practices are adopted.

In the absence of improvements in functional recovery, we believe the ergogenic benefits of SB-ORAL might be attributed to enhanced extracellular buffering capacity following oral SB given 3 d post. Immediately before the soccer-specific TB, blood pH and HCO_3_^−^ were elevated for SB-ORAL but not SB-LOTION compared with PLA (~0.05 au, 4–5 mmol·L^−1^). This supports previous research challenging whether PR Lotion is an effective delivery vehicle for SB [13,30]. Higher HCO_3_^−^ after oral SB likely permitted greater efflux of H^+^ from working muscles during high-intensity exercise, thus offsetting the decline in intramuscular pH that leads to allosteric inhibition of important glycolytic enzymes (e.g., phosphofructokinase-1, glycogen phosphorylase) [12]. Whilst not statistically significant, blood lactate was higher post-TB for SB-ORAL (~3 mmol·L^−1^), suggesting oral SB up-regulated glycolytic flux to some degree [17]. In contrast to pre-TB, blood pH and HCO_3_^−^ were significantly higher (~0.06 au, ~3 mmol·L^−1^) after exercise for SB-LOTION compared with PLA, despite a similar workload between conditions. This agrees with previous findings that PR Lotion protects blood pH during soccer-specific exercise [14]. Absorption of the SB molecules from PR Lotion could take longer than initially hypothesized, meaning the peak changes pre-TB were missed. There might also be a high degree of inter-individual variation for transdermal delivery rates of SB because of differences in skin structure [31]. Our investigation has highlighted several questions about PR Lotion, including (i) its purported induced cooling sensation hypothesis, (ii) absorption rates of SB across the skin, and (iii) optimal application timing to elicit ergogenic benefits. It would be beneficial for research to explore these questions in the future.

Neither time nor condition effects were shown for aggregate GI, suggesting that oral SB ingestion in a split-dose can be an ergogenic strategy for team sport athletes when evidence-based recommendations are followed. Negative connotations that prevent SB from being used might be attributed to studies using suboptimal supplementation approaches (e.g., fluid ingestion) [55]. Co-administering SB (in capsule form) with carbohydrate food mitigates GI symptoms—including abdominal discomfort and diarrhea—as it helps buffer stomach acid and slow down the absorption of HCO_3_^−^ that disturbs the GI system. Timing of SB also influences whether symptoms are detrimental. To best avoid side-effects, oral SB should be ingested ~2–2.5 h before exercise [56]. In contrast to Cameron et al. [55], who reported severe GI side-effects after prescribing SB 65 min pre–rugby-specific exercise, our supplementation timing (i.e., 2 h pre-TB) likely allowed full absorption of HCO_3_^−^ before participants began maximal performance tests. It should be noted, however, that split-dose timing approaches do not always eliminate side-effects [47]. Other strategies for mitigating GI distress after SB include progressive-chronic supplementation (e.g., 0.05 up to 0.2 g·kg^−1^ BM) and smaller doses (0.2 g·kg^−1^ BM) [57], or a novel hydrogel mini-tablet system [58], but their effectiveness in soccer requires further investigation.

This study has some limitations that should be addressed by future research. First, given the complex experimental protocol, it was difficult to retain participants (~20% dropped out) and meet our power calculation. Both male and female soccer players had to be included to achieve a sample close to our a priori calculation (*n* = 9 per condition), meaning sex-specific differences in anaerobic performance, with and without SB, may have influenced outcomes [38]. Best efforts were made to minimize this cofounding variable by matching the number of men and women receiving each condition and analyzing changes in performance relative to baseline (rather than comparing group averages 3 d post), but the inter-individual variability in responses would have been less with a homogeneous sample. Second, due to the applied nature of this study, it was not possible to use biochemical techniques to measure markers of muscle damage, cellular stress, and mitochondrial biogenesis (e.g., creatine kinase, HSP72, PGC-1α). Future work should adopt previous methodologies [19,23,51] to investigate whether SB attenuates cellular stress response and augments PGC-1α expression during/after EIMD. Due to the exploratory nature of this study and the athlete cohort available, we chose a valid and reliable EIMD task [26,45], rather than one that reflected the duration of a soccer game or matched the physical nature of contact sports (i.e., body collisions) [7,8]. Research examining the effect of SB during ecologically valid scenarios (e.g., between two soccer matches) is now warranted. Lastly, as a few participants identified SB, some of the reported improvements in soccer-specific performance for SB-ORAL might be explained by participants’ expectations that SB elicits ergogenic benefits, rather than its pharmacological effects [59].

## 5. Conclusions

Neither oral nor topical SB supplementation accelerated recovery after running-based muscle damage exercise, with functional and subjective indices of recovery similar to placebo up to 3 d post. Oral SB accelerated restoration of blood pH following EIMD, suggesting that underlying physiological benefits may exist requiring further investigation. Decline in soccer-specific performance (i.e., agility) from baseline to 3 d post was attenuated by administering oral SB again pre-TB, which was likely attributed to elevated extracellular buffering capacity. Real-world implications of these findings are that oral ingestion of SB might be a worthwhile strategy for rescuing key indicators of performance in soccer players 3 d after muscle damaging exercise, but additional research is needed during ecologically valid scenarios to strengthen evidence-based nutritional practices.

## Figures and Tables

**Table 1:** Participant characteristics and descriptive statistics between the SB-ORAL (*n* = 8), SB-LOTION (*n* = 8), and PLA (*n* = 8) conditions.

Variable	SB-ORAL	SB-LOTION	PLA	*p* Value
Sex (M/F)	5 M, 3 F	5 M, 3 F	6 M, 2 F	-
Body mass (kg)	76.8 ± 12.5	73.6 ± 7.0	81.4 ± 11.9	0.359
Stature (cm)	176.9 ± 11.1	176.1 ± 8.8	181.3 ± 8.3	0.505
Age (y)	21.1 ± 2.2	23.2 ± 2.6	22.0 ± 4.0	0.395

Values are mean ± SD. *p* value denotes one-way ANOVA outcome.

**Table 2:** Baseline dependent variables (functional capacity, performance) for each condition.

Variable	SB-ORAL	SB-LOTION	PLA	*p* Value
Soreness (mm)	49 ± 36	36 ± 21	36 ± 31	0.644
CMJ height (cm)	33.8 ± 6.9	34.1 ± 8.0	33.7 ± 5.2	0.993
DJ height (cm)	31.2 ± 4.6	32.6 ± 6.1	32.6 ± 5.9	0.843
MVIC (nm)	196 ± 74	183 ± 49	212 ± 51	0.634
IAT (s)	17.05 ± 0.92	17.18 ± 1.13	17.13 ± 1.02	0.967
8 × 25 m sprints (s)	4.04 ± 0.35	4.04 ± 0.26	4.04 ± 0.16	0.999
Yo-Yo IR2 (m)	425 ± 254	435 ± 220	425 ± 181	0.995

Values are mean ± SD. Muscle soreness is presented as an aggregate score out of 400 mm (quadriceps and gastrocnemius). *p* value denotes one-way ANOVA outcome.

**Table 3:** Repeated sprint performance during the EIMD task for each condition.

Variable	SB-ORAL	SB-LOTION	PLA	*p* Value
40 × 15 m sprints				
Average (s)	2.80 ± 0.18	2.81 ± 0.24	2.85 ± 0.12	0.872
Fastest (s)	2.65 ± 0.18	2.68 ± 0.19	2.70 ± 0.15	0.885
Total (s)	112.07 ± 7.43	112.73 ± 9.44	114.11 ± 4.72	0.857
Decrement (%)	5.6 ± 1.8	5.1 ± 2.2	5.9 ± 3.2	0.764

Values are mean ± SD. Decrement calculated as total time divided by fastest time multiplied by number of sprints and expressed as a percentage. *p* value denotes alpha statistic from one-way ANOVA.

**Table 4:** Time course responses for acid–base balance (pH, HCO_3_^−^).

Variable	Pre-EIMD	Post-EIMD	1 d Post	2 d Post	3 d Post	Pre- TB	Post-TB
pH (au)							
SB-ORAL	7.41 ± 0.02	7.38 ± 0.05	7.41 ± 0.01 *	7.41 ± 0.02	7.40 ± 0.02	7.46 ± 0.03 **	7.32 ± 0.05 *
SB-LOTION	7.40 ± 0.03	7.39 ± 0.02	7.39 ± 0.02	7.40 ± 0.03	7.39 ± 0.03	7.40 ± 0.01	7.31 ± 0.03 *
PLA	7.41 ± 0.02	7.37 ± 0.04	7.39 ± 0.01	7.40 ± 0.02	7.40 ± 0.02	7.41 ± 0.02	7.25 ± 0.07
HCO_3_^−^ (mmol·L^−1^)							
SB-ORAL	24.1 ± 1.3	20.4 ± 3.6	24.5 ± 1.8	23.6 ± 1.6	23.8 ± 2.2	28.7 ± 2.7 **	16.9 ± 2.4
SB-LOTION	24.2 ± 1.9	20.9 ± 2.1	24.1 ± 1.0	24.4 ± 2.2	23.9 ± 1.4	25.5 ± 1.9	17.3 ± 2.6 *
PLA	24.3 ± 1.3	20.5 ± 2.0	24.0 ± 1.7	24.0 ± 1.7	23.7 ± 1.1	23.8 ± 1.8	14.0 ± 2.7

Values are mean ± SD. TB = testing battery. Symbols depict significance: * vs. PLA (*p* < 0.05), ** vs. SB-LOTION and PLA (*p* < 0.05).

**Table 5:** Aggregate scores for gastrointestinal distress and cooling.

Variable	Post- EIMD	Post- Supp-1	Pre- Supp-4	Post- Supp-4	Pre-TB	Post-TB
GI (mm)						
SB-ORAL	-	-	23 ± 25	24 ± 23	20 ± 17	29 ± 28
SB-LOTION	-	-	11 ± 14	19 ± 33	14 ± 24	22 ± 25
PLA	-	-	19 ± 17	21 ± 17	22 ± 11	25 ± 23
Cooling (au)						
SB-ORAL	1 ± 1	−2 ± 2	0 ± 1	−2 ± 2	0 ± 1	2 ± 2
SB-LOTION	1 ± 1	−2 ± 2	0 ± 2	−2 ± 3	−1 ± 2	2 ± 2
PLA	1 ± 1	−2 ± 2	0 ± 1	−2 ± 1	−1 ± 2	3 ± 3

Values are mean ± SD. Aggregate GI distress (out of 800 mm) calculated from sum of eight visual analogue scales. Aggregate cooling sensations were quantified from summing ratings for the quadriceps and calves. TB = testing battery.

## Data Availability

The data presented in this study are available on request from the corresponding author. The data are not publicly available due to ethical restrictions.

## Abbreviations

The following abbreviations are used in this manuscript:

BM	Body mass
CMJ	Countermovement jump
DJ	Drop jump
EIMD	Exercise-induced muscle damage
GI	Gastrointestinal
H^+^	Hydrogen ion
HCO_3_^−^	Bicarbonate
HSP72	Heat shock protein-72
IAT	Illinois Agility Test
ICC	Intraclass correlation coefficient
MVIC	Maximal voluntary isometric contraction
PGC-1α	Peroxisome proliferator-activated receptor-γ coactivator 1-alpha
SB	Sodium bicarbonate
TB	Testing battery
TEM	Typical error of measurement
TRPM8	Transient receptor potential melastatin-eight
Yo-Yo IR2	Yo-Yo Intermittent Recovery Test Level 2
